# *o*-Toluene­sulfonamide: a redetermination

**DOI:** 10.1107/S1600536809033686

**Published:** 2009-08-29

**Authors:** B. Thimme Gowda, Sabine Foro, K. Shakuntala, Hartmut Fuess

**Affiliations:** a Department of Chemistry, Mangalore University, Mangalagangotri 574 199, Mangalore, India; b Institute of Materials Science, Darmstadt University of Technology, Petersenstrasse 23, D-64287 Darmstadt, Germany

## Abstract

The structure of the title compound, C_7_H_9_NO_2_S, was previously determined from powder diffraction data [Tremayne, Seaton & Glidewell (2002). *Acta Cryst.* B**58**, 823–834]. It has now been refined to a significantly higher precision. The amino N-atom is bent with a C—C—S—N torsion angle of −65.8 (2)deg;. In the crystal, mol­ecules are packed into a three-dimensional framework/supramolecular structure through hydrogen bonds between the two H atoms of the sulfonamide group and sulfonyl O atoms of neighbouring mol­ecules.

## Related literature

For our studies of the effect of substituents on the solid state structures of sulfonamides, see: Gowda *et al.* (2003 ▶, 2009 ▶); Gowda, Srilatha *et al.* (2007 ▶). For the parent benzene­sulfonamide, see: Gowda, Nayak *et al.* (2007 ▶). For other aryl sulfonamides, see: Gowda *et al.* (2003 ▶, 2009 ▶); Gowda, Srilatha *et al.* (2007 ▶); Jones & Weinkauf (1993 ▶); Kumar *et al.* (1992 ▶); O’Connor & Maslen (1965 ▶). For the powder structure of the title compound, see: Tremayne *et al.* (2002 ▶).
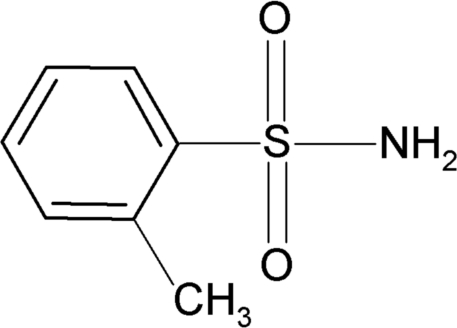


## Experimental

### 

#### Crystal data

C_7_H_9_NO_2_S
*M* *_r_* = 171.21
Tetragonal, 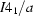

*a* = 18.670 (3) Å
*c* = 9.057 (1) Å
*V* = 3157.0 (8) Å^3^
*Z* = 16
Cu *K*α radiation
μ = 3.24 mm^−1^
*T* = 299 K
0.40 × 0.35 × 0.02 mm

#### Data collection

Enraf–Nonius CAD-4 diffractometer
Absorption correction: ψ scan (North *et al.*, 1968 ▶) *T*_min_ = 0.324, *T*_max_ = 0.938
5320 measured reflections
1403 independent reflections
1290 reflections with *I* > 2σ(*I*)
*R*_int_ = 0.059
3 standard reflections frequency: 120 min intensity decay: 1.5%

#### Refinement

*R*[*F* ^2^ > 2σ(*F* ^2^)] = 0.039
*wR*(*F* ^2^) = 0.101
*S* = 1.07
1403 reflections
108 parameters
2 restraints
H atoms treated by a mixture of independent and constrained refinement
Δρ_max_ = 0.28 e Å^−3^
Δρ_min_ = −0.41 e Å^−3^

### 

Data collection: *CAD-4-PC* (Enraf–Nonius, 1996 ▶); cell refinement: *CAD-4-PC*; data reduction: *REDU4* (Stoe & Cie, 1987 ▶); program(s) used to solve structure: *SHELXS97* (Sheldrick, 2008 ▶); program(s) used to refine structure: *SHELXL97* (Sheldrick, 2008 ▶); molecular graphics: *PLATON* (Spek, 2009 ▶); software used to prepare material for publication: *SHELXL97*.

## Supplementary Material

Crystal structure: contains datablocks I, global. DOI: 10.1107/S1600536809033686/fl2262sup1.cif

Structure factors: contains datablocks I. DOI: 10.1107/S1600536809033686/fl2262Isup2.hkl

Additional supplementary materials:  crystallographic information; 3D view; checkCIF report

## Figures and Tables

**Table 1:** Hydrogen-bond geometry (Å, °)

*D*—H⋯*A*	*D*—H	H⋯*A*	*D*⋯*A*	*D*—H⋯*A*
N1—H11⋯O2^i^	0.839 (16)	2.193 (18)	3.003 (2)	162 (2)
N1—H12⋯O1^ii^	0.841 (16)	2.138 (17)	2.964 (2)	167 (2)

Symmetry codes: (i) 
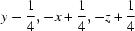
; (ii) 
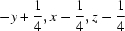
.

**Fig. 1.**
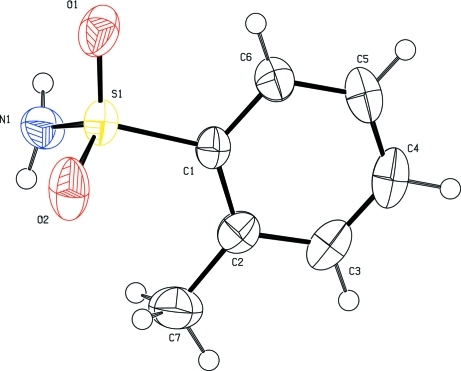
Molecular structure of (I), showing the atom labelling scheme and displacement ellipsoids are drawn at the 50% probability level.

**Fig. 2.**
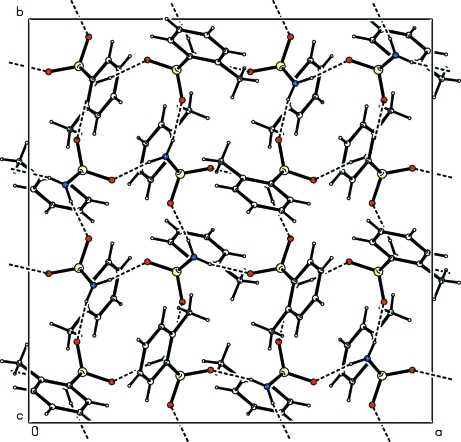
Molecular packing of (I) with hydrogen bonding shown as dashed lines.

## supplementary crystallographic information

### Comment

The chemistry of sulfonamides is of interest as they show distinct physical, chemical and biological properties. Many arylsulfonamides exhibit pharmacological, fungicidal and herbicidal activities. In the present work, the structure of (I) has been determined as part of our work to explore the effect of substituents on the solid state structures of sulfonamides (Gowda *et al.*, 2003, 2009; Gowda, Srilatha *et al.*, 2007).

The structure of (I) solved from X-ray powder data has been reported (Tremayne *et al.*, 2002) and the present single-crystal X-ray study confirms the powder diffraction structural parameters. (I)  crystallizes in the tetragonal I 41/a space group, in contrast to the monoclinic *Pc* space group observed with the parent benzenesulfonamide (BSA)(Gowda, Nayak *et al.*, 2007), the monoclinic cc space group for 2-chlorobenzenesulfonamide (2CBSA)(Gowda *et al.*, 2009), the orthorhombic Pbca space group for both 4-fluorobenzenesulfonamide (Jones & Weinkauf, 1993) and 4-aminobenzenesulfonamide (O'Connor & Maslen, 1965), and the monoclinic P21/n space group for both 4-chlorobenzenesulfonamide and 4-bromobenzenesulfonamide (Gowda *et al.*, 2003), and 4-methylbenzenesulfonamide (Kumar *et al.*, 1992). The orientation of the amino group with respect to the ring is given by the C–C–S–N torsional angle of -65.8 (2)°, compared to the values of -78.1 (10)° for BSA and 64.0 (2)° for 2CBSA. In (I), the molecules are packed into layers paralel to the *b*-axis through N1—H11···O1(S) and N1—H12···O2(S) intermolecular hydrogen bonding (Table 1 & Fig.2).

### Experimental

The purity of the commmercial sample (TCI, Tokyo) was checked and characterized by its infrared spectra. The single crystals used in X-ray diffraction studies were grown from ethanol by a slow evaporation of the solvent at room temperature.

### Refinement

The H atoms of the NH_2_ group were located in difference map and refined with restrained geometry to 0.86 (2) Å. The other H atoms were positioned with idealized geometry using a riding model [C—H = 0.93–0.96 Å]. All H atoms were refined with isotropic displacement parameters (set to 1.2 times of the *U*_eq_ of the parent atom).

### Crystal data

**Table:**

C_7_H_9_NO_2_S	*D*_x_ = 1.441 Mg m^−^^3^
*M**_r_* = 171.21	Cu *K*α radiation, λ = 1.54180 Å
Tetragonal, *I*4_1_/*a*	Cell parameters from 25 reflections
Hall symbol: -I 4ad	θ = 4.7–17.9°
*a* = 18.670 (3) Å	µ = 3.24 mm^−^^1^
*c* = 9.057 (1) Å	*T* = 299 K
*V* = 3157.0 (8) Å^3^	Prism, colourless
*Z* = 16	0.40 × 0.35 × 0.02 mm
*F*(000) = 1440	

### Data collection

**Table:**

Enraf–Nonius CAD-4 diffractometer	1290 reflections with *I* > 2σ(*I*)
Radiation source: fine-focus sealed tube	*R*_int_ = 0.059
graphite	θ_max_ = 66.8°, θ_min_ = 4.7°
ω/2θ scans	*h* = −22→22
Absorption correction: ψ scan (North *et al.*, 1968)	*k* = −22→22
*T*_min_ = 0.324, *T*_max_ = 0.938	*l* = −10→0
5320 measured reflections	3 standard reflections every 120 min
1403 independent reflections	intensity decay: 1.5%

### Refinement

**Table:**

Refinement on *F*^2^	Secondary atom site location: difference Fourier map
Least-squares matrix: full	Hydrogen site location: inferred from neighbouring sites
*R*[*F*^2^ > 2σ(*F*^2^)] = 0.039	H atoms treated by a mixture of independent and constrained refinement
*wR*(*F*^2^) = 0.101	*w* = 1/[σ^2^(*F*_o_^2^) + (0.0529*P*)^2^ + 2.2812*P*] where *P* = (*F*_o_^2^ + 2*F*_c_^2^)/3
*S* = 1.07	(Δ/σ)_max_ = 0.001
1403 reflections	Δρ_max_ = 0.28 e Å^−^^3^
108 parameters	Δρ_min_ = −0.41 e Å^−^^3^
2 restraints	Extinction correction: *SHELXL97* (Sheldrick, 2008), Fc^*^=kFc[1+0.001xFc^2^λ^3^/sin(2θ)]^-1/4^
Primary atom site location: structure-invariant direct methods	Extinction coefficient: 0.00227 (18)

### Special details

**Table:**

Geometry. All e.s.d.'s (except the e.s.d. in the dihedral angle between two l.s. planes) are estimated using the full covariance matrix. The cell e.s.d.'s are taken into account individually in the estimation of e.s.d.'s in distances, angles and torsion angles; correlations between e.s.d.'s in cell parameters are only used when they are defined by crystal symmetry. An approximate (isotropic) treatment of cell e.s.d.'s is used for estimating e.s.d.'s involving l.s. planes.
Refinement. Refinement of *F*^2^ against ALL reflections. The weighted *R*-factor *wR* and goodness of fit *S* are based on *F*^2^, conventional *R*-factors *R* are based on *F*, with *F* set to zero for negative *F*^2^. The threshold expression of *F*^2^ > σ(*F*^2^) is used only for calculating *R*-factors(gt) *etc*. and is not relevant to the choice of reflections for refinement. *R*-factors based on *F*^2^ are statistically about twice as large as those based on *F*, and *R*- factors based on ALL data will be even larger.

### Fractional atomic coordinates and isotropic or equivalent isotropic displacement parameters (Å^2^)

**Table:**

	*x*	*y*	*z*	*U*_iso_*/*U*_eq_	
S1	0.13337 (2)	0.12556 (2)	0.26312 (5)	0.0365 (2)	
O1	0.20600 (8)	0.10124 (10)	0.24990 (15)	0.0543 (5)	
O2	0.11947 (10)	0.20074 (8)	0.25836 (16)	0.0612 (5)	
N1	0.09068 (10)	0.08933 (10)	0.13117 (18)	0.0454 (4)	
H11	0.0510 (10)	0.1073 (12)	0.108 (3)	0.054*	
H12	0.1053 (12)	0.0484 (10)	0.107 (3)	0.054*	
C1	0.10065 (9)	0.09226 (9)	0.43325 (19)	0.0329 (4)	
C2	0.03095 (11)	0.10630 (10)	0.4810 (2)	0.0395 (5)	
C3	0.01179 (12)	0.07885 (12)	0.6185 (2)	0.0522 (5)	
H3	−0.0341	0.0873	0.6542	0.063*	
C4	0.05868 (14)	0.03960 (13)	0.7032 (2)	0.0575 (6)	
H4	0.0441	0.0222	0.7946	0.069*	
C5	0.12671 (13)	0.02595 (13)	0.6539 (2)	0.0545 (6)	
H5	0.1583	−0.0007	0.7113	0.065*	
C6	0.14805 (11)	0.05211 (11)	0.5179 (2)	0.0430 (5)	
H6	0.1940	0.0429	0.4832	0.052*	
C7	−0.02347 (13)	0.14733 (13)	0.3939 (3)	0.0609 (6)	
H7A	−0.0344	0.1218	0.3047	0.073*	
H7B	−0.0046	0.1937	0.3695	0.073*	
H7C	−0.0663	0.1529	0.4515	0.073*	

### Atomic displacement parameters (Å^2^)

**Table:**

	*U*^11^	*U*^22^	*U*^33^	*U*^12^	*U*^13^	*U*^23^
S1	0.0422 (3)	0.0384 (3)	0.0289 (3)	−0.00894 (17)	−0.00042 (16)	0.00272 (16)
O1	0.0375 (8)	0.0865 (12)	0.0388 (8)	−0.0106 (7)	0.0015 (6)	0.0054 (7)
O2	0.1032 (14)	0.0364 (8)	0.0441 (9)	−0.0154 (8)	0.0039 (8)	0.0066 (6)
N1	0.0483 (10)	0.0500 (10)	0.0378 (9)	0.0088 (8)	−0.0116 (7)	−0.0078 (7)
C1	0.0401 (10)	0.0305 (8)	0.0281 (8)	−0.0057 (7)	−0.0005 (7)	−0.0006 (7)
C2	0.0427 (11)	0.0357 (10)	0.0401 (10)	−0.0035 (8)	0.0019 (8)	−0.0061 (7)
C3	0.0541 (13)	0.0591 (13)	0.0435 (11)	−0.0101 (10)	0.0151 (9)	−0.0081 (9)
C4	0.0769 (16)	0.0612 (14)	0.0345 (10)	−0.0178 (12)	0.0063 (10)	0.0060 (10)
C5	0.0673 (15)	0.0549 (13)	0.0413 (12)	−0.0066 (10)	−0.0086 (10)	0.0160 (9)
C6	0.0445 (11)	0.0439 (11)	0.0405 (10)	−0.0014 (8)	−0.0025 (8)	0.0056 (8)
C7	0.0474 (12)	0.0632 (14)	0.0721 (16)	0.0123 (11)	0.0017 (11)	0.0027 (12)

### Geometric parameters (Å, °)

**Table:**

S1—O2	1.4281 (16)	C3—C4	1.375 (4)
S1—O1	1.4349 (16)	C3—H3	0.9300
S1—N1	1.5877 (17)	C4—C5	1.370 (3)
S1—C1	1.7703 (17)	C4—H4	0.9300
N1—H11	0.839 (16)	C5—C6	1.384 (3)
N1—H12	0.841 (16)	C5—H5	0.9300
C1—C6	1.390 (3)	C6—H6	0.9300
C1—C2	1.396 (3)	C7—H7A	0.9600
C2—C3	1.394 (3)	C7—H7B	0.9600
C2—C7	1.497 (3)	C7—H7C	0.9600
			
O2—S1—O1	118.70 (11)	C2—C3—H3	119.0
O2—S1—N1	107.74 (10)	C5—C4—C3	120.5 (2)
O1—S1—N1	106.07 (9)	C5—C4—H4	119.8
O2—S1—C1	107.98 (9)	C3—C4—H4	119.8
O1—S1—C1	106.72 (8)	C4—C5—C6	119.4 (2)
N1—S1—C1	109.41 (9)	C4—C5—H5	120.3
S1—N1—H11	117.3 (17)	C6—C5—H5	120.3
S1—N1—H12	114.9 (17)	C5—C6—C1	119.9 (2)
H11—N1—H12	126 (2)	C5—C6—H6	120.1
C6—C1—C2	121.59 (17)	C1—C6—H6	120.1
C6—C1—S1	116.72 (15)	C2—C7—H7A	109.5
C2—C1—S1	121.69 (14)	C2—C7—H7B	109.5
C3—C2—C1	116.55 (19)	H7A—C7—H7B	109.5
C3—C2—C7	119.0 (2)	C2—C7—H7C	109.5
C1—C2—C7	124.44 (19)	H7A—C7—H7C	109.5
C4—C3—C2	122.1 (2)	H7B—C7—H7C	109.5
C4—C3—H3	119.0		
			
O2—S1—C1—C6	−128.04 (16)	S1—C1—C2—C7	2.5 (3)
O1—S1—C1—C6	0.61 (17)	C1—C2—C3—C4	−0.3 (3)
N1—S1—C1—C6	114.96 (15)	C7—C2—C3—C4	178.8 (2)
O2—S1—C1—C2	51.23 (17)	C2—C3—C4—C5	−0.2 (3)
O1—S1—C1—C2	179.89 (15)	C3—C4—C5—C6	0.1 (4)
N1—S1—C1—C2	−65.77 (17)	C4—C5—C6—C1	0.4 (3)
C6—C1—C2—C3	0.9 (3)	C2—C1—C6—C5	−0.9 (3)
S1—C1—C2—C3	−178.38 (14)	S1—C1—C6—C5	178.33 (17)
C6—C1—C2—C7	−178.2 (2)		

### Hydrogen-bond geometry (Å, °)

**Table:**

*D*—H···*A*	*D*—H	H···*A*	*D*···*A*	*D*—H···*A*
N1—H11···O2^i^	0.84 (2)	2.19 (2)	3.003 (2)	162 (2)
N1—H12···O1^ii^	0.84 (2)	2.14 (2)	2.964 (2)	167 (2)

Symmetry codes: (i) *y*−1/4, −*x*+1/4, −*z*+1/4; (ii) −*y*+1/4, *x*−1/4, *z*−1/4.
